# Mechanism of Inhibiting the Growth and Aflatoxin B_1_ Biosynthesis of *Aspergillus flavus* by Phenyllactic Acid

**DOI:** 10.3390/toxins15060370

**Published:** 2023-06-01

**Authors:** Chi Zhao, Petri Penttinen, Lingzi Zhang, Ling Dong, Fengju Zhang, Zhihua Li, Xiaoping Zhang

**Affiliations:** 1 College of Resources, Sichuan Agricultural University, 211 Huimin Rd., Chengdu 611130, China; zhaochizc@163.com (C.Z.);; 2 Institute of Agro-Products Processing Science and Technology, Sichuan Academy of Agricultural Sciences, 60 Shizishan Rd., Chengdu 610066, China; 3 Faculty of Agriculture and Forestry, University of Helsinki, Viikinkaari 1, 00014 Helsinki, Finland

**Keywords:** phenyllactic acid, *Aspergillus flavus*, aflatoxin B_1_, transcriptome, metabolome

## Abstract

Phenyllactic acid (PLA), a promising food preservative, is safe and effective against a broad spectrum of food-borne pathogens. However, its mechanisms against toxigenic fungi are still poorly understood. In this study, we applied physicochemical, morphological, metabolomics, and transcriptomics analyses to investigate the activity and mechanism of PLA inhibition of a typical food-contaminating mold, *Aspergillus flavus*. The results showed that PLA effectively inhibited the growth of *A. flavus* spores and reduced aflatoxin B_1_ (AFB_1_) production by downregulating key genes associated with AFB_1_ biosynthesis. Propidium iodide staining and transmission electron microscopy analysis demonstrated a dose-dependent disruption of the integrity and morphology of the *A. flavus* spore cell membrane by PLA. Multi-omics analyses showed that subinhibitory concentrations of PLA induced significant changes in *A. flavus* spores at the transcriptional and metabolic levels, as 980 genes and 30 metabolites were differentially expressed. Moreover, KEGG pathway enrichment analysis indicated PLA-induced cell membrane damage, energy-metabolism disruption, and central-dogma abnormality in *A. flavus* spores. The results provided new insights into the anti-*A. flavus* and -AFB_1_ mechanisms of PLA.

## 1. Introduction

Mycotoxin contamination in foodstuff threatens human health and causes great economic losses every year [1]. According to the United Nations Food and Agricultural Organization (FAO), approximately one-quarter of agricultural products are affected by mycotoxins [1]. Approximately 450 million people in developing countries experience long-term exposure to large doses of mycotoxins [2,3]. Multiple mycotoxins are present in the food chain, as contamination can occur at every stage (cultivation, storage, processing, and transportation) of food production [4]. Numerous toxins, e.g., aflatoxins, ochratoxins, fumonisins, zearalenone, patulin, enniatins, Alternaria toxins, and trichothecenes, are frequently encountered in food systems [4]. Among them, aflatoxin B_1_ (AFB_1_), produced by *Aspergillus flavus*, is the most important and hazardous mycotoxin [5]. The AFB_1_ was classified as a group I human carcinogen because it was strongly teratogenic, carcinogenic, hepatotoxic, and immunosuppressive to the health of humans [6]. Therefore, efficient control of *A. flavus* infections and AFB_1_ contamination has become an important research topic.

Currently, AFB_1_ contamination produced by *A. flavus* is controlled using physical, chemical, and biological methods [7]. In particular, chemical fungicides are effective and economical tools to control *A. flavus* infections and AFB_1_ contamination [8]. However, excessive and non-selective use of chemical fungicides promotes drug resistance and may result in toxic residues that pose a serious threat to human health [9]. Therefore, there is an urgent need to find efficient and non-toxic natural anti-fungal compounds to replace or complement traditional chemical fungicides to improve food safety and shelf life [8]. Phenyllactic acid (PLA) is a natural phenolic acid commonly found in honey and traditional fermented foods that exhibits broad-spectrum and efficient inhibitory activity against food-borne bacterial and fungal pathogens [10,11]. In addition, PLA is safe and has good water solubility and no objectionable odor, making it a promising preservative for the food industry [12,13]. Therefore, the anti-microbial mechanism of PLA has attracted attention. Ning et al. (2017) reported dual anti-bacterial targets of PLA were the cell membrane and genomic DNA. When *Bacillus cereus* was exposed to PLA, proteomic analysis showed that its K+ transport was inhibited, leading to the dissipation of the membrane potential and disruption of ribosome function [14]. In a recent study, combining transcriptomics and metabolomics revealed that PLA acted against *Rhizopus oryzae* by inhibiting energy metabolism [15]. Moreover, previous studies had demonstrated that PLA effectively inhibits the growth and AFB_1_ biosynthesis of *A. flavus* [16,17]. However, the mechanism of inhibition has not been reported.

In this study, we investigated the activity of PLA to inhibit *A. flavus* and AFB_1_ and revealed its inhibition mechanism by physicochemical, morphological, metabolomic, and transcriptomic analyses. The results provided new insights into the effects of PLA against *A. flavus* and AFB_1_.

## 2. Results

### 2.1. Anti-A. flavus and -AFB_1_ Activity of PLA

**Figure 1:**
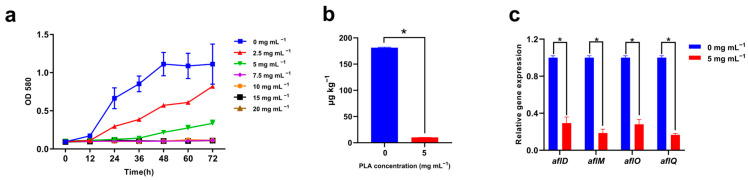
Effects of phenyllactic acid on *Aspergillus flavus* (**a**) growth, (**b**) aflatoxin B_1_ production, and (**c**) relative expression levels of aflatoxin B_1_ biosynthesis-related genes. The error bars indicate the standard deviation (*n* = 3). * represents statistical significance at *p* < 0.05.

In a potato dextrose broth (PDB) medium, the growth of *A. flavus* spores was inhibited by 2.5 and 5 mg mL^−1^ concentrations of PLA, and no growth was detected at PLA concentrations ≥7.5 mg mL^−1^ (Figure 1a). In addition, no *A. flavus* spores were detected on the potato dextrose agar (PDA) with a PLA ≥ 10 mg mL^−1^. Thus, the minimum inhibitory concentration (MIC) and minimum fungicidal concentration (MFC) of PLA against *A. flavus* spores were 7.5 and 10 mg mL^−1^, respectively. In determining the time-dependent fungicidal activity of PLA, the number of viable spores remained relatively stable at a ≤2.5 mg mL^−1^ concentration of PLA (Table 1). At 5 and 7.5 mg mL^−1^ PLA, the numbers of viable spores decreased from 5 to 15 min and from 15 to 30 min (*p* < 0.05). At 10, 15, and 20 mg mL^−1^ PLA, the *A. flavus* spores were completely inactivated after 120, 15, and 5 min, respectively. 

PLA effectively inhibited the production of AFB_1_ by *A. flavus*, and the results showed that the concentration of AFB_1_ in the supernatant of the treatment group (5 mg mL^−1^ PLA) was 10.20 ± 0.03 μg L^−1^, which was significantly lower (*p* < 0.05) than that of the control group (181.20 ± 1.08 μg L^−1^) (Figure 1b). The result of quantitative real-time polymerase chain reaction (qRT-PCR) analysis showed that the relative expressions of four key genes related to AFB_1_ biosynthesis were also significantly (*p* < 0.05) downregulated after treatment with PLA (Figure 1c). 

### 2.2. Effects of PLA on Cell Membrane Integrity and Morphology of A. flavus Spores

**Figure 2:**
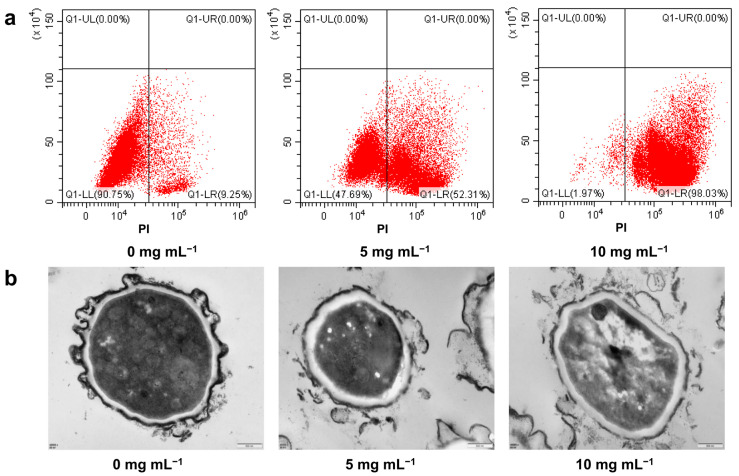
(**a**) Flow cytometry dot plots and (**b**) transmission electron microscopy images of *Aspergillus flavus* spores treated with 0 mg mL^−1^, 5 mg mL^−1^ or 10 mg mL^−1^ phenyllactic acid for 30 min.

After a 30-minute incubation in a PDB medium with 0, 5, or 10 mg mL^−1^ PLA, the cell membranes of 8.59 ± 0.62%, 54.26 ± 1.88%, and 98.02 ± 0.01% of *A. flavus* spores were disrupted, respectively (Figure 2a). In addition, TEM examination showed that PLA damaged the *A. flavus* spore structure in a dose-dependent manner, resulting in the loss of cytoplasm and abnormal cell morphology (Figure 2b). 

### 2.3. Global Analysis of The Transcriptomic Response and qRT-PCR Validation

In this study, approximately 42,000,000 clean reads were obtained from each treatment, and about 91% of clean reads were mapped to the *A. flavus* NRRL3357 genome (Supplementary Table S1). The raw data have been deposited at the National Microbiology Data Center (NMDC) with the accession number NMDC40037319.

**Figure 3:**
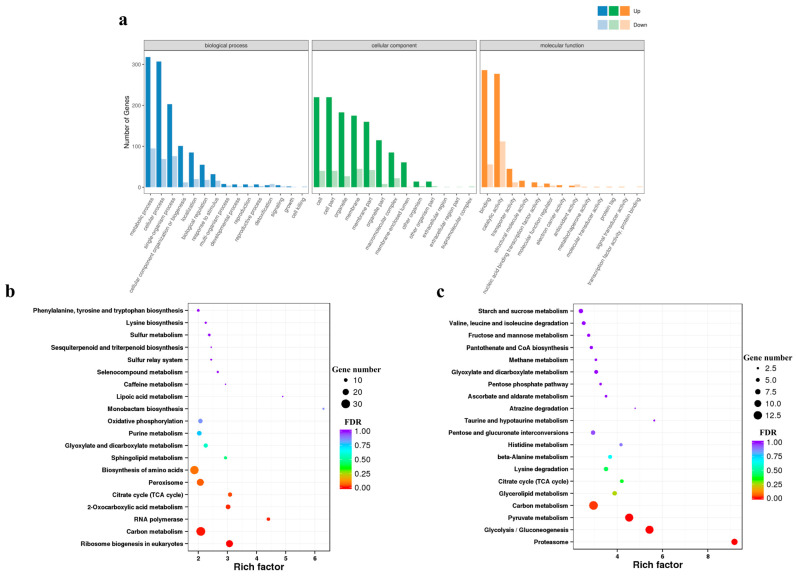
Functional and pathway analysis of differentially expressed genes. (**a**) Functional categorization of differentially expressed genes in GO. KEGG enrichment analysis of (**b**) up- and (**c**) down-regulated differentially expressed genes.

Comparative transcriptomic results showed that the treatment of *A. flavus* spores with PLA resulted in changes at the mRNA level (Supplementary Figure S1). In comparison to the control group, a total of 980 genes were differentially expressed (|log2 (fold change)| > 1, false discovery rate (FDR) < 0.05) in the PLA group, of which 711 differentially expressed genes (DEGs) were upregulated and 269 DEGs were downregulated. The functional categories of DEGs were annotated through GO enrichment analysis and the results are shown in Figure 3a. DEGs are grouped into three categories: biological process, cellular component, and molecular function, consisting of 15, 13, and 13 subcategories, respectively. Within the biological process category, metabolic processes, cellular processes, single-organism processes, and cellular component organization or biogenesis were the main subcategories distributed by DEGs. The cell, cell part, organelle, and membrane were the main subcategories within the cellular component categories. While for the molecular function category, the top four subcategories by frequency were binding, catalytic activity, transporter activity, and structural molecule activity. 

The functions of DEGs were further annotated using KEGG enrichment analysis. The results indicated that upregulated DEGs were mainly (FDR < 0.05) involved in the pathways of ribosome biogenesis in eukaryotes, carbon metabolism, RNA polymerase, 2-oxocarboxylic acid metabolism, and the citrate cycle (TCA cycle) (Figure 3b). However, PLA treatment downregulated DEGs mainly (FDR < 0.05) associated with proteasome, glycolysis/gluconeogenesis, pyruvate metabolism, and carbon metabolism (Figure 3c). To validate the results of the transcriptome, four DEGs (AFLA_130070, *HK*; AFLA_037480, *ENO*; AFLA_049290, *CS*; AFLA_098420, *SDHA*) were analyzed by qRT-PCR. Among them, *HK* and *ENO* were downregulated in the PLA treatment group, whereas *CS*, *SDHA*, *Erg25*, *Erg5*, *TTDA,* and *ERCC3* were upregulated, which was consistent with the RNA-Seq results (Supplementary Figure S2).

### 2.4. Global Analysis of The Metabolomic Response 

A total of 59 metabolites were detected, including lipids and lipid-like molecules (21), organic acids and derivatives (21), organic oxygen compounds (7), organoheterocyclic compounds (5), benzenoids (4), and organic nitrogen compounds (1) (Table 2).

**Figure 4:**
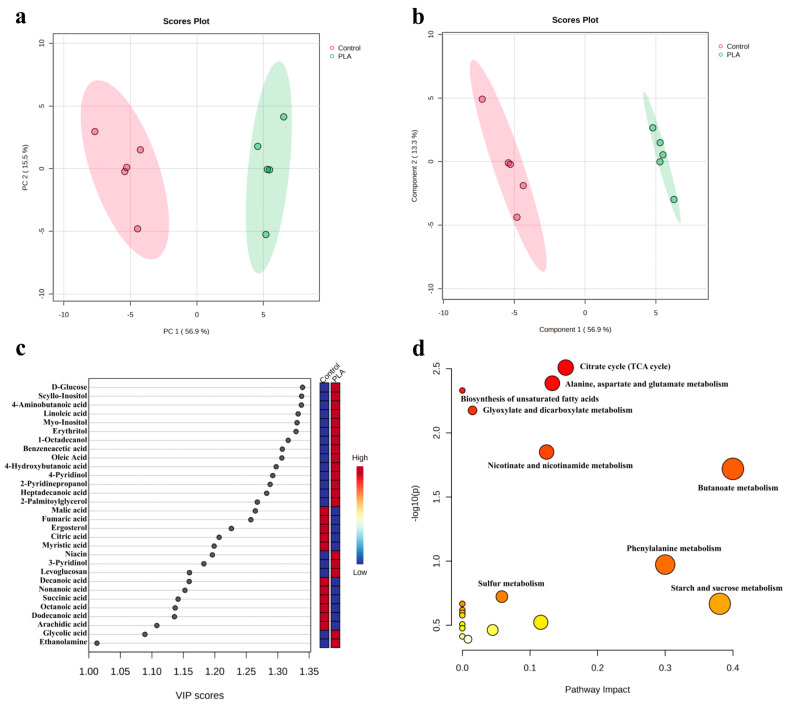
Influence of PLA on metabolomic profiles of *Aspergillus flavus*. (**a**) PCA and (**b**) PLS-DA of the metabolites detected by GC-MS in *A. flavus* treated with or without phenyllactic acid. (**c**) Variable importance of the projection (VIP) values of metabolites with VIP > 1 in the PLS-DA model. (**d**) KEGG pathway analysis of differential metabolites.

In the principal component analysis (PCA; PC1: 56.9% and PC2: 15.5%), the samples treated with or without PLA were completely separated, with the five biological replicates grouped in the same cluster, suggesting differences between the control and PLA treatments (Figure 4a). According to the partial least squares discrimination analysis (PLS-DA) model (Q2 = 0.949, R2 = 0.980), 30 out of the detected 59 metabolites were differential with variable importance of the projection (VIP) > 1 (Figure 4b). Compared to the control, the peak intensities of 19 differential metabolites (DMs) were higher in the PLA treatment and those of 11 lower (Figure 4c). The Kyoto Encyclopedia of Genes and Genome (KEGG) enrichment analysis was carried out to annotate the DMs into different metabolic pathways, mainly including the citrate cycle (TCA cycle), alanine, aspartate, and glutamate metabolism, the biosynthesis of unsaturated fatty acids, glyoxylate, and dicarboxylate metabolism, nicotinate and nicotinamide metabolism and butanoate metabolism (Figure 4d and Supplementary Table S2).

## 3. Discussion

We studied applying PLA, a promising preservative for the food industry, against AFB_1_ producing *A. flavus* CICC 2219. In line with the MFC of PLA against food-borne *Aspergillus species* [16,18], the MIC and MFC of PLA against *A. flavus* spores were 7.5 and 10 mg mL^−1^. In addition, the inactivating activity of PLA against *A. flavus* spores was time- and dose-dependent. Compared to lactic acid, a traditional food-preserving organic acid, PLA had been reported to have a more rapid inactivation of *Enterobacter cloacae* and *Listeria monocytogenes* [11,19,20]. AFB_1_ is the most dangerous secondary metabolite biosynthesized by *A. flavus* and has caused serious health and economic problems [2]. Previous studies have reported that PLA can effectively inhibit the biosynthesis of aflatoxins, and has stronger anti-aflatoxin activity compared to hydroxyphenyllactic acid, indole lactic acid, and lactic acid [17]. In this study, the finding that PLA has been shown to have efficient inhibitory activity on the growth and AFB_1_ biosynthesis of *A. flavus* was again confirmed in vitro. In addition, qRT-PCR was used to further investigate the molecular mechanism of AFB_1_ reduction under PLA stress, and it was found that the expression of four key genes related to AFB_1_ biosynthesis (*aflD*, *aflM*, *aflQ,* and *aflR*) was significantly downregulated (*p* < 0.05) after PLA treatment [21]. These results demonstrated for the first time that PLA reduces the concentration of AFB_1_ biosynthesis in *A. flavus* by downregulating the expression of genes related to AFB_1_ biosynthesis, which is similar to the results of previous studies [22,23].

The cell membrane plays an important role in the cell structure and physiological function [24]. Based on the results of propidium iodide (PI) staining and transmission electron microscopy (TEM) analysis, the cell membrane was one of the anti-*A. flavus* spore targets of PLA. Previous studies had demonstrated that PLA is able to damage the cell membrane of bacteria, resulting in bacteriostatic activity [11,12,19]. In this study, the cytoplasmic loss of *A. flavus* spores after PLA treatment may also be caused by damage to the cell membrane, similar to the mechanism of bacterial inhibition by PLA. In addition, changes related to the cell membrane have also been found at the level of transcription and metabolism. Firstly, ABC transporters are a superfamily of widely distributed membrane proteins that transport substances across the cell membrane [25]. In this study, the genes encoding the ABC multidrug transporters (AFLA_064360, *ABCB1*; AFLA_036800, *SNQ2*) were upregulated in the PLA treatment group, suggesting that PLA may affect the normal transport function of the *A. flavus* spore cell membrane [26]. Similarly, Lei et al. reported that trans-anethole inhibited *A. flavus* and upregulated the expression of its ABC multidrug transporters genes [27]. Likewise, when *Candida albicans* was exposed to *Huanglian Jiedu*, which has antifungal activity, the transcriptome results showed a significant upregulation of ABC multidrug transporters gene expression [28]. Secondly, fatty acids are important components of cell membranes with biological and structural functions, especially unsaturated fatty acids, which have an important impact on cell membrane permeability and fluidity [29,30]. Luz et al. (2014) reported that *Origanum vulgare* L. essential oil damaged the cell membranes of *Salmonella typhimurium*, and induced an increase in unsaturated fatty acids at sublethal concentrations [31]. In this study, the KEGG pathway analysis of DMs showed that the biosynthesis of unsaturated fatty acids was enriched, and the peak intensities of unsaturated fatty acids (linoleic acid and oleic acid) were higher in the PLA treatment group. In addition, in the results of transcriptome analysis, the expression of genes (AFLA_004970, *ELO3*; AFLA_019690, *ACAA1*) associated with unsaturated fatty-acid synthesis were all increased in the PLA treatment group compared to the control group. Thirdly, ergosterol is an essential sterol component of fungal cell membranes and plays an important role in maintaining cell-membrane integrity and cell function [32]. Therefore, the ergosterol biosynthesis pathway is an important target for many fungicides [28]. For example, Xu et al. (2021) found that cuminaldehyde inhibited *A. flavus* by decreasing the relative content of ergosterol and downregulating the expression of genes related to the ergosterol synthesis pathway [32]. Similarly, the peak intensity of ergosterol in *A. flavus* spores decreased after PLA treatment in this study. In contrast, the relative expression levels of all genes (AFLA_110370, *Erg6*; AFLA_115530, *Erg25*, AFLA_116730, *FDFT1*; AFLA_039610, *Erg6*; AFLA_028640, *Erg5*) associated with ergosterol biosynthesis were upregulated after PLA treatment. This may be a response to PLA damage to the cell membrane of *A. flavus*, which was similar to the findings of Yang et al. (2016) [28].

**Figure 5:**
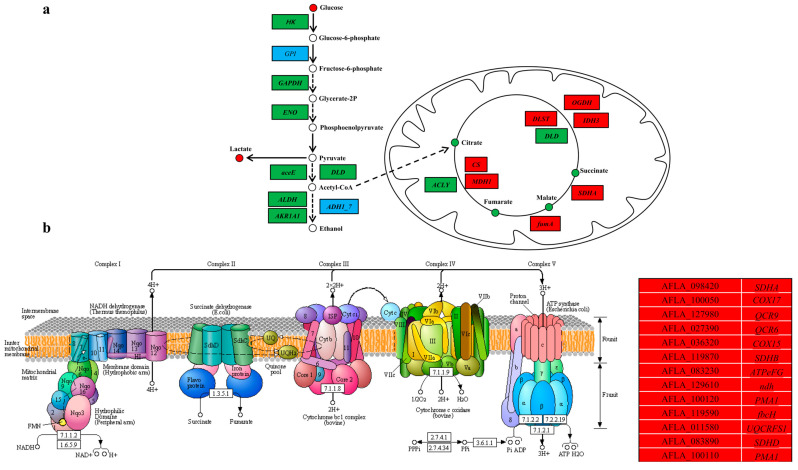
KEGG enrichment analysis of differentially expressed genes and differential metabolites associated with (**a**) glycolysis and TCA cycle, and (**b**) oxidative phosphorylation. Squares and circles represent differentially expressed genes (DEGs) and differential metabolites (DMs), respectively. The red, green, and blue in the squares or circles represent the upregulation, downregulation, and up as well as downregulation of DEGs and DMs, respectively.

Energy metabolism is an essential metabolic pathway in microbial growth and development [33]. In a recent study, a proteomic analysis showed that PLA could disrupt energy metabolism to inhibit *R. oryzae* [15]. Similarly, KEGG enrichment analysis of DMs and DEGs in this study revealed enrichment of some metabolic pathways related to energy metabolism, including pyruvate metabolism, oxidative phosphorylation, glycolysis/gluconeogenesis, and the citrate cycle (TCA cycle), suggesting that PLA also influences energy metabolism in *A. flavus* spores. Glucose is one of the essential substrates of energy metabolism, which is converted to pyruvate via glycolysis and finally enters the TCA cycle [34]. In this study, the peak intensity of glucose increased in the PLA treatment group, whereas the transcriptome results showed that the expression levels of most genes associated with glycolysis were downregulated (Figure 5a). This indicated that PLA may inhibit the expression of glycolysis-related genes, resulting in the accumulation of glucose. Moreover, PLA decreased the peak intensities of citric acid, malic acid, succinic acid, and fumaric acid, significant intermediates in the TCA cycle, and the results were consistent with the findings of Fan et al. (2022) [15]. However, the transcriptomic results were the opposite, with the expression levels of most genes related to the TCA cycle being upregulated, probably due to the different concentration and time of the PLA treatment as well as different inhibitory microorganisms (Figure 5a). In addition, oxidative phosphorylation is an important part of energy metabolism [35]. In this study, the relative expression of all DEGs associated with oxidative phosphorylation was upregulated after treatment with PLA (Figure 5b). Overall, PLA disrupted the energy metabolism in *A. flavus* spores.

Ling et al. (2017) reported that bacterial genomic DNA was able to interact with PLA in vitro; presumably, DNA was one of the inhibitory targets of PLA [12]. In this study, the expression of a DNA replication-related gene (AFLA_045970, *MCM4*), encoding the DNA replication licensing factor MCM4, was found to be significantly downregulated after PLA treatment (Table 3). MCM proteins are key components of the pre-replication complex and may play a role in the development of replication forks as well as the recruitment of other DNA replication-related proteins [36]. Among them, MCM4 is a highly conserved mini-chromosome maintenance protein that is essential for the start of eukaryotic genome replication [37]. This suggested that PLA may affect DNA replication. Similarly, Yao et al. (2019) reported that DNA replication in *Zygosaccharomyces rouxii* was inhibited by acids produced by *Tetragenococcus halophilus* [35].

Moreover, 16 genes involved in transcription were detected as DEGs, and notably, their relative expression levels were all increased in the PLA treatment group (Table 3). AFLA_060250 and AFLA_006070 encode TFIIH basal transcription factor complex TTDA subunit and DNA excision repair protein ERCC3, respectively, both of which are components of the multi-subunit transcription/repair factor IIH (TFIIH) complex and affect basal transcription [38,39]. RNA polymerase is the essential enzyme for transcription and its transfers of genetic information from DNA to mRNA, while the RNA transport process, transferring a variety of RNA from the nucleus to the cytoplasm, all have an important impact on the transcription process [35,40]. Notably, the relative expression levels of all DEGs related to RNA polymerase (AFLA_017530, *RPA1*; AFLA_017150, *RPABC1*; AFLA_048730, *RPA49*; AFLA_030070, *RPABC2*; AFLA_071240, *RPA43*; AFLA_129630, *RPA12*; AFLA_055410, *RPAC2*; AFLA_037100, *RPA2*; AFLA_137020, *RPAC1*) and RNA transport (AFLA_086940, *EIF4A*; AFLA_118010, *EIF4E*; AFLA_083090, *rnz*; AFLA_046010, *EIF5*; AFLA_018110, *NUP93*) were also increased in the PLA treatment group. Moreover, for protein synthesis (spliceosome, aminoacyl-tRNA biosynthesis, ribosome, ribosome biogenesis in eukaryotes, and protein export), a total of 51 genes were identified as DGEs. Among them, only 3 genes (AFLA_117970, *IARS*; AFLA_034320, *NUG1*; AFLA_066480, *XRN1*) showed downregulation of relative expression levels after PLA treatment, while the rest of the genes were upregulated. Overall, these results showed for the first time that the processes of central dogma may be an important target of the PLA effect in *A. flavus*, and further research on key genes will be conducted in the future.

## 4. Conclusions

In this study, the anti-*A. flavus* and -AFB_1_ activity and mechanism of PLA were investigated by physicochemical, morphological, metabolomic, and transcriptomic analyses. The results showed that PLA effectively inhibited and inactivated *A. flavus*, and decreased AFB_1_ production by downregulating key genes associated with AFB_1_ biosynthesis. In addition, combined transcriptomic and metabolomic analyses showed that PLA induced multiple effects such as cell-membrane damage, energy-metabolism disruption, and central-dogma abnormality. The results provide new insights into the activity and mechanisms of PLA in inhibiting toxigenic fungi.

## 5. Materials and Methods

### 5.1. Reagents, Media, and Fungal Strain

PLA, isopropanol, acetonitrile, methoxyamine hydrochloride, pyridine, and N-methyl-N-(trimethylsilyl)trifluoroacetamide with 1% trimethylchlorosilane were purchased from Sigma-Aldrich Co. (Shanghai, China). Czapek-Dox agar, PDB, and PDA were purchased from Haibo Co. (Qingdao, China).

*A. flavus* CICC 2219, a standard strain for the identification of aflatoxin-producing *A. flavus*, was obtained from the China Center of Industrial Culture Collection (CICC, Beijing, China). The strain was cultivated on Czapek-Dox agar at 28 °C for 7 days to prepare spore suspension in sterile water. The spore suspension was adjusted to 10^7^ spores mL^−1^ using a hemocytometer.

### 5.2. Determination of Minimum Inhibitory and Minimum Fungicidal Concentrations

In a previous study, the MFC of PLA against *A. flavus* was 7.5–10 mg mL^−1^, indicating that the inhibitory activity of PLA on *A. flavus* is strain-specific [16]. Therefore, the MIC and MFC of PLA against *A. flavus* CICC 2219 were determined by a previously described method with slight modifications [16]. 

Briefly, 80 μL of the PDB medium, 20 μL of *A. flavus* spore suspension (10^7^ spores mL^−1^), and 100 μL of various concentrations of PLA solution were mixed in a 96-well microliter plate to make a final concentration of PLA at 0, 2.5, 5, 7.5, 10, 15 and 20 mg mL^−1^, respectively. Then, the plate was incubated in the dark at 28 °C, and the growth of *A. flavus* was monitored at 12 h intervals for 3 days using a multimode plate reader (Synergy™ HTX, BioTek Instruments, Winooski, VT, USA). The MIC was defined as the lowest concentration of PLA that completely inhibited growth. To determine MFC, 50 μL of spore suspension was spread on PDA plates containing PLA at concentrations ≥ MIC and incubated at 28 °C for 72 h. The MFC was defined as the lowest concentration of PLA that resulted in colony-free plates.

### 5.3. Fungicidal Activity of PLA over Time

The 30 μL of *A. flavus* spore suspension (10^7^ spores mL^−1^) and 4970 μL of various concentrations of PLA solution were mixed in a 10 mL centrifuge tube to make the final concentration of PLA at 0, 2.5, 5, 7.5, 10, 15, and 20 mg mL^−1^, respectively. Then, at 5, 15, 30, 60, and 120 min, 50 μL of the 0–10^5^ fold diluted suspensions were spread on PDA plates. The plates were incubated at 28 °C for 3 days, and colonies were counted.

### 5.4. Effects of PLA on AFB1 Content and Biosynthesis-Related Genes

The effects of PLA on AFB_1_ content and biosynthesis-related genes were analyzed as described previously with a slight modification [22]. Briefly, 30 μL of spore suspension (10^7^ spores mL^−1^) was inoculated into 30 mL of the PDB medium containing 0 and 5 mg mL^−1^ PLA, and incubated at 180 rpm and 28 °C for 4 days. Subsequently, the suspension was centrifuged (12,000 rpm, 10 min, 4 °C), and the supernatant was collected. The AFB^1^ concentration in the supernatant was determined by isotope dilution liquid chromatography-tandem mass spectrometry (ID-LC-MS/MS) according to the Chinese national standards (GB 5009.22-2016).

The total RNA of mycelia was extracted using an RNAprep Pure Plant Kit (Tiangen Biotech Co., Ltd., Beijing, China). The quality and quantity of RNA were determined using a NanoDrop 2000 spectrophotometer (Thermo Fisher Scientific, Wilmington, DE, USA) and an Agilent 2100 Bioanalyzer (Agilent Technologies, Santa Clara, CA, USA). Then, cDNA was synthesized using a PrimeScript RT reagent Kit (Takara Biotechnology Co., Ltd., Dalian, China). Four key genes (*aflD*, *aflM*, *aflO,* and *aflQ*) related to AFB_1_ biosynthesis were amplified using the primers listed in Supplementary Table S3. Twenty μL qRT-PCR contained 10 μL of 2×Real PCR EasyTM Mix-SYBR, 0.8 μL of forward and reverse primer (10 μM), 2 μL of cDNA, and 6.4 μL of ddH_2_O water. The qRT-PCR was carried out using a QuantStudio TM3 Real-Time PCR System (Thermo Fisher Technology Co., Ltd., Wilmington, DE, USA), and the procedure was consistent with the description of Xu et al. (2021) [22]. Data were analyzed using the 2^−ΔΔCT^ method in Thermo Scientific PikoReal.

### 5.5. Cell Membrane and Spore Integrity

Briefly, 100 μL of spore suspension (10^7^ spores mL^−1^) was inoculated into 30 mL of PDB with 0, 5, and 10 mg mL^−1^ PLA. After a 30-minute incubation at 28 °C in the dark, the spores were collected by centrifugation at 1500 rpm and 4 °C for 5 min. The spore pellets were washed 3 times with PBS and resuspended in 500 μL of PBS. 

The integrity of the cell membrane of *A. flavus* spores was determined using PI staining. Briefly, 5 μL of PI and spore re-suspension were mixed and incubated in the dark for 15 min and then detected using a Beckman Coulter Cytoflex S flow cytometer (Beckman Coulter, CA, USA). The results were analyzed using CytExpert v 2.3 software.

The integrity of *A. flavus* spores was determined using TEM. The suspensions were centrifuged at 1500 rpm for 10 min, the supernatant was removed, and spores were fixed with 2.5% (*v*/*v*) glutaraldehyde. The fixed spore specimens were dehydrated, embedded, sectioned, stained, and examined using a JEM-1400PLUS transmission electron microscope (JEOL, Tokyo, Japan).

### 5.6. Transcriptomic Analysis and qRT-PCR Validation

Fifteen mL of the spore suspension (10^7^ spores mL^−1^) was inoculated into 15 mL of the PLA solution to make the final concentration of PLA in the treatment group. The concentration was 2.5 mg mL^−1^, and incubated at 180 rpm and 28 °C for 30 min. Moreover, the treatment with sterile water instead of PLA solution was used as the control group. Subsequently, the spores of *A. flavus* were collected (8000 rpm, 10 min, 4 °C) for transcriptomic and qRT-PCR analysis. 

The total RNA was extracted using an RNAprep Pure Plant Kit (Tiangen Biotech Co., Ltd., Beijing, China). The quality and quantity of RNA in the extracts were determined using a NanoDrop 2000 spectrophotometer (Thermo Fisher Scientific, Wilmington, DE, USA) and an Agilent 2100 Bioanalyzer (Agilent Technologies, Santa Clara, CA, USA). Subsequently, cDNA library preparation was carried out using the NEBNext Ultra TM RNA Library Prep Kit for Illumina (NEB, Ipswich, MA, USA), and sequencing by the Illumina platform in Biomarker Technologies Co. (Beijing, China). Post-analysis used the BMKCloud (www.biocloud.net; accessed on 22 February 2022) online bioinformatics pipeline tool platform. Briefly, clean data was obtained by processing raw data using in-house perl scripts. Then, transcriptomic data was mapped and assembled based on the *A. flavus* NRRL3357 genome using HISAT2 (version 2.2.1) and StringTie (version 2.0) software, respectively [41,42]. The FPKM (fragments per kilobase per transcript per million mapped reads) value was used to express the expression abundance of the corresponding genes. The calculation of the false discovery rate (FDR) was to correct the *p*-value. Differentially expressed genes (DEGs) were identified by DEseq2, with |log2 (fold change)| > 1 and FDR < 0.05 taken as conditions [43]. Gene function was annotated using the KO (Kyoto Encyclopedia of Genes and Genome Orthologue) and GO (Gene Ontology) databases. The GO and the Kyoto Encyclopedia of Genes and Genome (KEGG) enrichment analyses were carried out using the GOseq R packages and KOBAS software, respectively [44,45].

Furthermore, qRT-PCR was carried out to validate the results on the expression levels of key DEGs (*HK*, *ENO*, *CS*, *SDHA*, *Erg25*, *Erg5*, *TTDA*, *ERCC3*) associated with glycolysis and TCA cycle in the transcriptome (Supplementary Table S3). The cDNA synthesis kit, qRT-PCR reaction system, and instrumentation were the same as in Section 5.4. The procedure for qRT-PCR consisted of pre-denaturation at 95 °C for 30 s, 45 cycles of denaturation at 95 °C for 5 s, annealing at 55 °C for 30 s, elongation at 72 °C for 30 s, and final extension at 72 °C for 30 s, followed by an analysis of the data in Thermo Scientific PikoReal using the 2^−ΔΔCT^ method.

### 5.7. Metabolome Analysis

The treatments of *A. flavus* spores were the same as in Section 5.6, and the extraction and derivatization of intracellular metabolites in *A. flavus* spores were analyzed as described previously [46]. Briefly, the spores were collected by centrifugation at 8000 rpm and 4 °C for 2 min. The spore pellets were washed with sterile water, immediately frozen in liquid nitrogen, and ground into powder. Metabolites were extracted by dissolving 100 mg of spore powder into 1 mL of 3:2:2 isopropanol: acetonitrile: water (*v*/*v*/*v*) solution and shaking the solution for 2.5 min in an MM400 mixer mill (Retsch Technology, Haan, Germany). The solution was centrifuged at 12,000 rpm for 5 min, 200 μL of the supernatant was lyophilized, and the residue was methoximated by dissolving into 100 μL of 20 mg mL^−1^ methoxyamine hydrochloride in pyridine and incubating for 90 min in a 30 °C water bath. Silylation was carried out by adding 50 μL of N-methyl-N-(trimethylsilyl)trifluoroacetamide with 1% trimethylchlorosilane and incubating at 37 °C for 30 min.

Metabolites were analyzed using an Agilent Intuvo 9000-5977 B gas chromatography-mass spectrometer (GC-MS) system (Agilent Corporation, Santa Clara, CA, USA). Metabolites were separated in a 30 m × 0.25 mm × 0.25 µm HP-5 MS column (Agilent Corporation, Santa Clara, CA, USA) with a 1.0 mL min^−1^ helium flow rate. The injector, transfer line, and ion source temperatures were 250, 280, and 230 °C, respectively. The column temperature was 80 °C for 2 min, after which the temperature was increased from 10 °C min^−1^ to 300 °C, which was held for 6 min. The metabolites were scanned at *m*/*z* 50–600.

Peak detection and alignment were performed using MS-DIAL v 4.16 [47]. Compounds were identified using the National Institute of Standards and Technology (NIST) 17 database. The metabolites were classified by ClassyFire Batch (https://cfb.fiehnlab.ucdavis.edu/; accessed on 15 March 2023) [48]. PCA, PLS-DA, and KEGG pathway analyses were performed by the MetaboAnalyst (https://www.metaboanalyst.ca; accessed on 15 March 2023) webserver [49]. Metabolites with VIP > 1 in the PLS-DA model were considered as DMs.

### 5.8. Statistical Analysis

All experiments were performed at least in triplicate. Differences were tested using a one-way ANOVA and Duncan’s multiple range test in SPSS 22 (SPSS Inc., Chicago, IL, USA). Differences were taken as statistically significant at *p* < 0.05.

## Figures and Tables

**Table 1:** The numbers of viable *Aspergillus flavus* spores at different phenyllactic acid concentrations over time.

PLA (mg mL^−1^)	Viable Spores (10^4^ Spores mL^−1^)				
	5 min	15 min	30 min	60 min	120 min
0	11.67 ± 0.58 ^a^	12.00 ± 1.00 ^a^	11.67 ± 0.58 ^a^	11.67 ± 1.53 ^a^	10.33 ± 0.58 ^a^
2.5	11.33 ± 0.58 ^a^	12.67 ± 0.58 ^a^	12.00 ± 1.00 ^a^	10.67 ± 2.52 ^a^	10.67 ± 1.15 ^a^
5	7.67 ± 0.58 ^a^	4.67 ± 0.58 ^b^	2.33 ± 0.58 ^c^	1.33 ± 0.21 ^c^	1.57 ± 0.12 ^c^
7.5	7.33 ± 0.58 ^a^	3.57 ± 0.38 ^b^	1.23 ± 0.25 ^c^	1.03 ± 0.06 ^c^	0.53 ± 0.06 ^c^
10	2.17 ± 0.15 ^a^	1.50 ± 0.36 ^a^	0.14 ± 0.03 ^c^	0.02 ± 0.01 ^c^	ND
15	0.18 ± 0.02	ND	ND	ND	ND
20	ND	ND	ND	ND	ND

Values are mean ± SD (*n* = 3). Different letters within a row indicate statistically significant differences (*p* < 0.05). ND represents not detected.

**Table 2:** Intracellular metabolites detected by GC-MS in *Aspergillus flavus* treated with or without phenyllactic acid.

No.	Compounds	RT ^(1)^ (min)	Peak Intensities (10^5^)		VIP ^(2)^
			0 mg mL^−1^	2.5 mg mL^−1^	
	**Lipids and lipid-like molecules**				
1	Hexanoic acid	5.67	1.54 ± 0.77	0.80 ± 0.56	0.70
2	Heptanoic acid	6.91	0.45 ± 0.08	0.36 ± 0.06	0.74
3	4-Hydroxybutanoic acid	7.85	1.32 ± 0.12	2.40 ± 0.19	1.30
4	Octanoic acid	8.19	4.90 ± 1.20	2.45 ± 0.18	1.14
5	Nonanoic acid	9.44	7.86 ± 1.79	4.02 ± 0.25	1.15
6	Decanoic acid	10.66	10.07 ± 2.67	4.24 ± 0.35	1.16
7	Undecanoic acid	11.81	2.48 ± 0.09	2.61 ± 0.09	0.85
8	Dodecanoic acid	12.91	5.02 ± 0.92	3.15 ± 0.13	1.14
9	Azelaic acid	14.51	0.38 ± 0.04	0.48 ± 0.09	0.83
10	Myristic acid	14.99	5.57 ± 0.99	3.06 ± 0.12	1.20
11	Palmitic acid	16.73	97.44 ± 9.12	90.34 ± 4.39	0.65
12	Heptadecanoic acid	17.77	1.28 ± 0.04	1.64 ± 0.08	1.28
13	1-Octadecanol	17.87	0.83 ± 0.03	1.19 ± 0.05	1.32
14	Oleic acid	18.43	8.97 ± 0.54	12.99 ± 0.52	1.31
15	Stearic acid	18.63	41.96 ± 24.41	51.04 ± 3.94	0.36
16	Linoleic acid	18.72	2.81 ± 0.13	4.74 ± 0.14	1.33
17	Arachidic acid	20.22	1.49 ± 0.19	1.04 ± 0.15	1.11
18	2-Palmitoylglycerol	21.16	0.31 ± 0.01	0.42 ± 0.03	1.27
19	1-Monopalmitin	21.41	68.53 ± 41.99	104.90 ± 1.86	0.79
20	Glycerol monostearate	22.82	54.81 ± 1.76	53.95 ± 0.88	0.44
21	Ergosterol	26.29	6.32 ± 0.81	4.02 ± 0.06	1.23
	**Organic acids and derivatives**				
22	Lactic acid	5.57	21.86 ± 5.75	29.14 ± 11.82	0.54
23	Glycolic acid	5.74	2.29 ± 0.33	2.92 ± 0.14	1.09
24	L-Alanine	6.09	48.18 ± 27.50	66.46 ± 39.96	0.38
25	Oxalic acid	6.48	8.02 ± 1.38	16.97 ± 7.28	0.93
26	L-2-Aminobutyric acid	6.97	1.44 ± 0.87	1.85 ± 1.17	0.32
27	L-Proline	7.12	0.80 ± 0.42	0.88 ± 0.09	0.20
28	L-Valine	7.62	3.49 ± 1.27	6.05 ± 1.81	0.91
29	Urea	7.91	6.80 ± 0.35	5.64 ± 3.22	0.34
30	L-Threonine	8.61	9.65 ± 0.28	8.88 ± 2.32	0.34
31	Succinic acid	8.88	0.91 ± 0.08	0.75 ± 0.02	1.14
32	Fumaric acid	9.29	2.57 ± 0.28	1.63 ± 0.02	1.26
33	Serine	9.55	11.52 ± 1.69	8.88 ± 3.70	0.61
34	L-Aspartic acid	10.30	31.77 ± 0.81	39.64 ± 6.97	0.89
35	Malic acid	11.16	3.35 ± 0.24	2.47 ± 0.07	1.26
36	L-5-Oxoproline	11.56	19.11 ± 1.01	17.99 ± 5.23	0.22
37	4-Aminobutanoic acid	11.62	0.06 ± 0.14	2.61 ± 0.13	1.34
38	L-Asparagine	12.40	7.03 ± 1.68	4.86 ± 2.76	0.64
39	L-Glutamic acid	12.67	87.37 ± 4.71	72.29 ± 15.44	0.80
40	L-Glutamine	14.37	25.26 ± 13.29	13.02 ± 7.21	0.72
41	L-Ornithine	14.88	6.62 ± 3.40	2.22 ± 0.15	0.96
42	Citric acid	14.91	107.46 ± 14.80	68.91 ± 1.01	1.21
	**Organic oxygen compounds**				
43	Diethylene glycol	7.96	1.03 ± 0.06	1.23 ± 0.13	0.99
44	Erythritol	11.36	20.15 ± 0.44	26.85 ± 0.61	1.33
45	D-Glucitol	16.11	7.98 ± 0.24	7.79 ± 0.75	0.24
46	D-Mannitol	16.19	1.79 ± 1.04	1.24 ± 0.70	0.50
47	Scyllo-Inositol	16.35	0.95 ± 0.02	1.38 ± 0.02	1.34
48	D-Glucose	16.54	0.84 ± 0.12	3.84 ± 0.10	1.34
49	Myo-Inositol	17.59	7.30 ± 0.15	10.37 ± 0.28	1.33
	**Organoheterocyclic compounds**				
50	3-Pyridinol	6.57	1.54 ± 0.15	3.13 ± 0.66	1.18
51	4-Pyridinol	6.79	2.63 ± 0.13	3.52 ± 0.15	1.29
52	Niacin	8.62	0.55 ± 0.06	0.87 ± 0.11	1.20
53	2-Pyridinepropanol	9.62	3.69 ± 0.74	8.42 ± 0.80	1.29
54	Levoglucosan	13.70	0.43 ± 0.25	1.00 ± 0.14	1.16
	**Benzenoids**				
55	Benzoic acid	8.01	3.05 ± 0.15	3.32 ± 1.87	0.24
56	Benzeneacetic acid	8.66	ND	0.45 ± 0.07	1.31
57	Terephthalic acid	14.49	12.33 ± 2.20	8.66 ± 1.49	0.99
58	Bisphenol A	18.54	3.63 ± 0.50	3.01 ± 0.22	0.90
	**Organic nitrogen compounds**				
59	Ethanolamine	8.29	1.81 ± 0.29	2.38 ± 0.25	1.01

Note: ^(1)^ RT: retention time; ^(2)^ This value is calculated with the PLS-DA model.

**Table 3:** Differentially expressed genes associated with cDNA replication, RNA transcription, and protein synthesis.

Gene Description	log2FC	Gene ID
**DNA replication**		
DNA replication licensing factor/*MCM4*	−1.39	AFLA_045970
**Basal transcription factors**		
TFIIH basal transcription factor complex/*TTDA*	1.11	AFLA_060250
DNA excision repair protein/*ERCC3*	1.14	AFLA_006070
**RNA polymerase**		
DNA-directed RNA polymerase I subunit/*RPA1*	1.54	AFLA_017530
DNA-directed RNA polymerases I, II, and III subunit/*RPABC1*	1.08	AFLA_017150
DNA-directed RNA polymerase I subunit/*RPA49*	1.24	AFLA_048730
DNA-directed RNA polymerases I, II, and III subunit/*RPABC2*	1.13	AFLA_030070
DNA-directed RNA polymerase I subunit/*RPA43*	1.33	AFLA_071240
DNA-directed RNA polymerase I subunit/*RPA12*	1.12	AFLA_129630
DNA-directed RNA polymerases I and III subunit/*RPAC2*	1.27	AFLA_055410
DNA-directed RNA polymerase I subunit/*RPA2*	1.28	AFLA_037100
DNA-directed RNA polymerases I and III subunit/*RPAC1*	1.07	AFLA_137020
**RNA transport**		
translation initiation factor/*EIF4A*	1.39	AFLA_086940
translation initiation factor/*EIF4E*	1.09	AFLA_118010
ribonuclease/*rnz*	1.12	AFLA_083090
translation initiation factor/*EIF5*	1.25	AFLA_046010
nuclear pore complex protein/*NUP93*	1.19	AFLA_018110
**Spliceosome**		
heat shock 70 kDa protein/*HSPA1s*	2.22	AFLA_043390
ATP-dependent RNA helicase/*DDX5*	1.42	AFLA_043840
U4/U6 small nuclear ribonucleoprotein/*SNU13*	1.46	AFLA_130100
pre-mRNA-splicing factor ATP-dependent RNA helicase/*DHX16*	1.52	AFLA_006100
pre-mRNA-splicing factor ATP-dependent RNA helicase/*DHX16*	1.68	AFLA_047160
pre-mRNA-splicing factor ATP-dependent RNA helicase/*DHX15*	1.87	AFLA_132910
heat shock 70 kDa protein/*HSPA1s*	1.31	AFLA_012200
survival of motor neuron-related-splicing factor/*SMNDC1*	1.14	AFLA_129350
**Aminoacyl-tRNA biosynthesis**		
isoleucyl-tRNA synthetase/*IARS*	−1.64	AFLA_117970
tryptophanyl-tRNA synthetase/*WARS*	1.09	AFLA_054690
aspartyl-tRNA(Asn)/glutamyl-tRNA(Gln) amidotransferase subunit/*gatA*	1.1	AFLA_089240
isoleucyl-tRNA synthetase/*IARS*	1.71	AFLA_073060
**Ribosome**		
large subunit ribosomal protein/*L24e*	1.44	AFLA_133350
large subunit ribosomal protein/*L33*	1.07	AFLA_129150
large subunit ribosomal protein/*L14*	1.79	Aspergillus_flavus_newGene_2055
small subunit ribosomal protein/*S5*	1.06	AFLA_132790
small subunit ribosomal protein/*S17*	1.17	AFLA_034020
large subunit ribosomal protein/*L5e*	1.57	AFLA_018730
large subunit ribosomal protein/*L27*	1.08	AFLA_006810
large subunit ribosomal protein/*L17*	1.28	AFLA_021760
small subunit ribosomal protein/*S14*	1.18	AFLA_043320
small subunit ribosomal protein/*S6*	1.29	AFLA_067460
large subunit ribosomal protein/*L34*	1.07	AFLA_112240
large subunit ribosomal protein/*L19*	1.03	AFLA_098510
**Ribosome biogenesis in eukaryotes**		
nuclear GTP-binding protein/*NUG1*	−1.09	AFLA_034320
H/ACA ribonucleoprotein complex subunit/*NOP10*	1.26	AFLA_006780
U3 small nucleolar RNA-associated protein/*UTP18*	1.28	AFLA_130080
nuclear GTP-binding protein/*NUG2*	1.43	AFLA_110550
nucleolar GTP-binding protein/*NOG1*	1.48	AFLA_134410
N-acetyltransferase/*NAT10*	1.64	AFLA_029920
small nucleolar RNA-associated protein/*DIP2*	1.16	AFLA_080740
rRNA 2′-O-methyltransferase fibrillarin/*NOP1*	1.3	AFLA_016990
nucleolar protein/*NOP56*	1.32	AFLA_085280
5′–3′ exoribonuclease/*XRN1*	−1.51	AFLA_066480
U4/U6 small nuclear ribonucleoprotein/*SNU13*	1.46	AFLA_130100
H/ACA ribonucleoprotein complex subunit/*DKC1*	1.37	AFLA_048200
U3 small nucleolar RNA-associated protein/*UTP5*	1.06	AFLA_026620
nuclear GTP-binding protein/*NUG1*	1.31	AFLA_069120
nucleolar protein/*NOP4*	1.42	AFLA_113720
U3 small nucleolar ribonucleoprotein protein/*IMP4*	1.18	AFLA_018390
H/ACA ribonucleoprotein complex subunit/*NHP2*	1.57	AFLA_017830
U3 small nucleolar RNA-associated protein/*UTP15*	1.3	AFLA_042550
nonsense-mediated mRNA decay protein/*NMD3*	1.03	Aspergillus_flavus_newGene_82
ribosome biogenesis protein/*BMS1*	1.15	AFLA_136700
H/ACA ribonucleoprotein complex subunit/*GAR1*	1.52	AFLA_052540
U3 small nucleolar RNA-associated protein/*UTP13*	1.19	AFLA_112310
casein kinase II subunit/*CSNK2B*	1.29	AFLA_069840
nucleolar protein/*NOP58*	1.46	AFLA_020450
U3 small nucleolar ribonucleoprotein protein/*IMP3*	1.19	AFLA_046700
**Protein export**		
YidC/Oxa1 family membrane protein insertase/*yidC*	1.19	AFLA_006440
endoplasmic reticulum chaperone/*BiP*	1.07	AFLA_035620

## Data Availability

The raw transcriptome data have been deposited at the National Microbiology Data Center (NMDC) under the registration number NMDC40037319 (accessed on 11 April 2023).

## Supplementary Materials

The following supporting information can be downloaded at: https://www.mdpi.com/article/10.3390/toxins15060370/s1, Table S1: Summary of reads in *A. flavus* with or without PLA treatment; Table S2: KEGG enrichment of significantly differential metabolites; Table S3: Primers used for qRT-PCR; Table S4: Differentially expressed genes associated with cell membrane; Figure S1: Heat map of correlation between phenyllactic acid treatment group and control group; Figure S2: Expression of the eight selected genes in the phenyllactic acid treatment group and control group.
