# Supplementary material for: Mechanism of Inhibiting the Growth and Aflatoxin B1 Biosynthesis of Aspergillus flavus by Phenyllactic Acid
Source: Toxins (Basel). 2023 Jun 1;15(6):370. doi: 10.3390/toxins15060370 (PMC10304077; doi:10.3390/toxins15060370)
Supplement: Supplementary file 1 [file toxins-15-00370-s001.zip › toxins-2398717-supplementary.pdf]

Supplementary Table S1. Summary of reads in *A. flavus* with or without PLA treatment.

Sample	Total clean reads	Total mapped reads	Mapping percentage
Control group-1	43,847,164	40,506,949	92.38%
Control group-2	41,749,588	38,025,719	91.08%
Control group-3	47,763,666	43,236,943	90.52%
Treatment group-1	38,732,408	35,285,321	91.10%
Treatment group-2	40,380,076	37,625,796	93.18%
Treatment group-3	42,880,110	39,765,460	92.74%

Supplementary Table S2. KEGG enrichment of significantly differential metabolites.

Pathway	Total	Hits	FDR	-log(p)	Impact
Citrate cycle (TCA cycle)	20	3	0.11393	2.5085	0.15292
Alanine, aspartate and glutamate metabolism	22	3	0.11393	2.3862	0.1331
Biosynthesis of unsaturated fatty acids	23	3	0.11393	2.3296	0
Glyoxylate and dicarboxylate metabolism	26	3	0.12193	2.1752	0.01506
Nicotinate and nicotinamide metabolism	12	2	0.20602	1.8504	0.12469
Butanoate metabolism	14	2	0.23245	1.7188	0.4
Phenylalanine metabolism	7	1	1	0.97346	0.3
Sulfur metabolism	13	1	1	0.72362	0.05844
Tyrosine metabolism	15	1	1	0.66776	0
Starch and sucrose metabolism	15	1	1	0.66776	0.38071
Galactose metabolism	17	1	1	0.61967	0
Arginine biosynthesis	18	1	1	0.59797	0
Propanoate metabolism	19	1	1	0.57761	0
Inositol phosphate metabolism	22	1	1	0.52326	0.11609
Pyruvate metabolism	23	1	1	0.50705	0
Arginine and proline metabolism	25	1	1	0.477	0
Phosphatidylinositol signaling system	26	1	1	0.46305	0.04493
Steroid biosynthesis	30	1	1	0.41314	0
Glycerophospholipid metabolism	32	1	1	0.3912	0.00833

Supplementary Table S3. Primers used for qRT-PCR.

Genes	Primer (5'-3')	Description
Actin	F:ACGGTGTCGTCACAAACTGG<br>R:CGGTTGGACTTAGGGTTGATAG	House-keeping gene
HK	F:TGTACGACACAGTCAAGGGC<br>R:CCGCTGGTATATGCGACCAT	Hexokinase
ENO	F:CAACACAAGGCACATGGCTC<br>R:TTATTACTCGCCTGTCCCGT	Enolase
CS	F:GGGGTGACGATGTGATTGGT<br>R:CTCATCGGGATCAAGGACCG	Citrate synthase
SDHA	F:ACAAGGGAAGGGCAACAACA<br>R:CAGACAGGTTCCCACCCATC	Succinate dehydrogenase (ubiquinone) flavoprotein subunit
Erg25	F:ATATTCTCACGATTGGCTAC<br>R:TGGATTTCTCATGGTATAGG	Methylsterol monooxygenase
Erg5	F:ACAAGTTCGTCGTCATCGCATCC<br>R:CCATCCAAGAACACCCAGTTATCCTC	Sterol 22-desaturase
TTDA	F:CCTAGATGATGACCGCCACC<br>R:TGCGCGTCGTCAGTATAGTC	TFIIH basal transcription factor complex TTD-A subunit
ERCC3	F:GAAAGTACCGATCCGGCGAT<br>R:GCTGCCTGCTGAATAATGCC	DNA excision repair protein ERCC-3
aflD	F:CGCCTGAGGAGACGGTGTATT<br>R:CTGCCTTCAGCGACGGTTAG	AFB_1 biosynthesis
aflM	F:CCGTTTAGATGGCAAAGTGGC<br>R:TCACGGGAATGGGCGTAGTT	
aflQ	F:TAAGGCAGCGGAATACAAGCG<br>R:CAAGAGGATGGAAGGACGAGA	
aflR	F:GCAGTCAATGGAACACGGAAA<br>R:CCTGAAACGGTGGTAGTGG	

Supplementary Table S4. Differentially expressed genes associated with cell membrane.

Gene Description	log2FC	Gene ID
**ABC transporters**		
ATP-binding cassette/*ABCB1*	1.02	AFLA_064360
ATP-binding cassette/*SNQ2*	2.74	AFLA_036800
**Biosynthesis of unsaturated fatty acids**		
fatty acid elongase/*ELO3*	1.69	AFLA_004970
acetyl-CoA acyltransferase/*ACAA1*	1.83	AFLA_019690
**Steroid biosynthesis**		
sterol 24-C-methyltransferase/*Erg6*	4.27	AFLA_110370
methylsterol monooxygenase/*Erg25*	1.03	AFLA_115530
farnesyl-diphosphate farnesyltransferase/*FDFT1*	1.11	AFLA_116730
sterol 24-C-methyltransferase/*Erg6*	2.07	AFLA_039610
sterol 22-desaturase/*Erg5*	1.64	AFLA_028640
**Glycolysis / Gluconeogenesis**		
phosphoenolpyruvate carboxykinase/*pckA*	-1.79	AFLA_036370
glucose-6-phosphate isomerase/*GPI*	1.43	AFLA_044830
dihydrolipoamide dehydrogenase/*DLD*	-1.09	AFLA_051890
alcohol dehydrogenase/*adh*	1.22	AFLA_128700
alcohol dehydrogenase/*frmA*	-2.37	AFLA_006730
alcohol dehydrogenase/*AKR1A1*	-2.32	AFLA_021160
hexokinase/*HK*	-1.1	AFLA_130070
aldehyde dehydrogenase/*ALDH*	-2.32	AFLA_108790
pyruvate dehydrogenase E1 component alpha subunit/*PDHA*	-1.75	AFLA_035290
enolase/*ENO*	-1.48	AFLA_037480
aldehyde dehydrogenase/*ALDH*	-1.77	AFLA_111070
aldehyde dehydrogenase/*ALDH*	-1.69	AFLA_015350
alcohol dehydrogenase/*adh*	1.08	AFLA_133040
glucose-6-phosphate isomerase/*GPI*	-1.79	AFLA_044820
glyceraldehyde 3-phosphate dehydrogenase/*GAPDH*	-1.34	AFLA_025100
**Citrate cycle**		
phosphoenolpyruvate carboxykinase/*pckA*	-1.79	AFLA_036370
succinate dehydrogenase (ubiquinone) flavoprotein subunit/*SDHA*	1.39	AFLA_098420
isocitrate dehydrogenase/*IDH3*	1.83	AFLA_018850
isocitrate dehydrogenase/*IDH3*	1.72	AFLA_037910
dihydrolipoamide dehydrogenase/*DLD*	-1.09	AFLA_051890
2-oxoglutarate dehydrogenase E1 component/*OGDH*	1.77	Aspergillus_flavus_newGene_1134
pyruvate dehydrogenase E1 component alpha subunit/*PDHA*	-1.75	AFLA_035290
citrate synthase/*CS*	1.94	AFLA_049290
ATP citrate (pro-S)-lyase/*ACLY*	-2.23	Aspergillus_flavus_newGene_1691
succinate dehydrogenase (ubiquinone) iron-sulfur subunit/*SDHB*	1.05	AFLA_119870
malate dehydrogenase/*MDH1*	1.51	AFLA_076710
dihydrolipoamide succinyltransferase/*DLST*	1.32	AFLA_104010
ATP citrate (pro-S)-lyase/*ACLY*	-2.44	AFLA_106350

citrate synthase/*CS*	1.81	AFLA_007020
fumarate hydratase/*fumA*	1.11	AFLA_091270
2-oxoglutarate dehydrogenase E1 component/*OGDH*	1.95	Aspergillus_flavus_newGene_1135
**Oxidative phosphorylation**		
succinate dehydrogenase (ubiquinone) flavoprotein subunit/*SDHA*	1.39	AFLA_098420
cytochrome c oxidase assembly protein subunit 17/*COX17*	1.85	AFLA_100050
ubiquinol-cytochrome c reductase subunit 9/*QCR9*	1.15	AFLA_127980
ubiquinol-cytochrome c reductase subunit 6/*QCR6*	1.18	AFLA_027390
heme a synthase/*COX15*	1.76	AFLA_036320
succinate dehydrogenase (ubiquinone) iron-sulfur subunit/*SDHB*	1.05	AFLA_119870
F-type H+-transporting ATPase subunit g/*ATPeFG*	1.01	AFLA_083230
quinone reductase (non-electrogenic)/*ndh*	1.67	AFLA_129610
H+-transporting ATPase/*PMA1*	2.95	AFLA_100120
ubiquinol-cytochrome c reductase cytochrome b/c1 subunit/*fbcH*	1.22	AFLA_119590
ubiquinol-cytochrome c reductase iron-sulfur subunit/*UQCRFS1*	1.14	AFLA_011580
succinate dehydrogenase (ubiquinone) membrane anchor subunit/*SDHD*	1.02	AFLA_083890
H+-transporting ATPase/*PMA1*	3.26	AFLA_100110

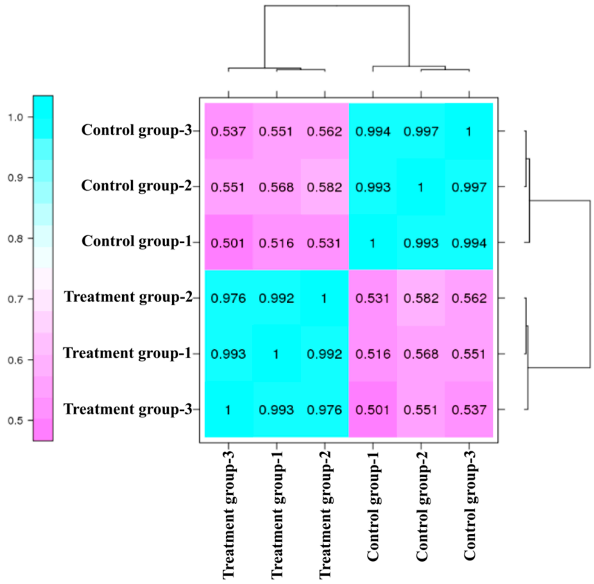

Supplementary Figure S1. Heat map of correlation between phenyllactic acid treatment group and control group. The number in the box represents the Pearson's correlation coefficient.

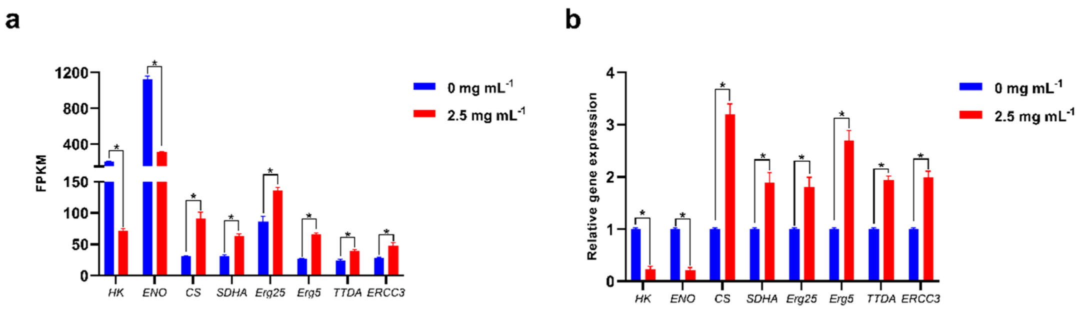

Supplementary Figure S2. Expression of the eight selected genes in the phenyllactic acid treatment group and control group. (a) Transcriptome results and (b) qRT-PCR results. * represents statistical significance at $P < 0.05$. FPKM=fragments per kilobase per transcript per million mapped reads.
